# Variable parallelism in the genomic basis of age at maturity across spatial scales in Atlantic Salmon

**DOI:** 10.1002/ece3.11068

**Published:** 2024-04-05

**Authors:** Tony Kess, Sarah J. Lehnert, Paul Bentzen, Steven Duffy, Amber Messmer, J. Brian Dempson, Jason Newport, Christopher Whidden, Martha J. Robertson, Gerald Chaput, Cindy Breau, Julien April, Carole‐Anne Gillis, Matthew Kent, Cameron M. Nugent, Ian R. Bradbury

**Affiliations:** ^1^ Northwest Atlantic Fisheries Centre Fisheries and Oceans Canada St. John's Newfoundland and Labrador Canada; ^2^ Department of Biology Dalhousie University Halifax Nova Scotia Canada; ^3^ Marine Environmental Research Infrastructure for Data Integration and Application Network Halifax Nova Scotia Canada; ^4^ Department of Computer Science Dalhousie University Halifax Nova Scotia Canada; ^5^ Fisheries and Oceans Canada Gulf Fisheries Centre Moncton New Brunswick Canada; ^6^ Ministère des Forêts de la Faune et des Parcs Quebec Quebec Canada; ^7^ Gespe'gewa'gi, Mi'gma'qi, Listuguj Gespe'gewa'gi Institute of Natural Understanding Quebec Quebec Canada; ^8^ Centre for Integrative Genetics Norwegian University of Life Sciences Ås Norway

**Keywords:** Atlantic Salmon, genetic architecture, genomics, life history

## Abstract

Complex traits often exhibit complex underlying genetic architectures resulting from a combination of evolution from standing variation, hard and soft sweeps, and alleles of varying effect size. Increasingly, studies implicate both large‐effect loci and polygenic patterns underpinning adaptation, but the extent that common genetic architectures are utilized during repeated adaptation is not well understood. Sea age or age at maturation represents a significant life history trait in Atlantic Salmon (*Salmo salar*), the genetic basis of which has been studied extensively in European Atlantic populations, with repeated identification of large‐effect loci. However, the genetic basis of sea age within North American Atlantic Salmon populations remains unclear, as does the potential for a parallel trans‐Atlantic genomic basis to sea age. Here, we used a large single‐nucleotide polymorphism (SNP) array and low‐coverage whole‐genome resequencing to explore the genomic basis of sea age variation in North American Atlantic Salmon. We found significant associations at the gene and SNP level with a large‐effect locus (*vgll3*) previously identified in European populations, indicating genetic parallelism, but found that this pattern varied based on both sex and geographic region. We also identified nonrepeated sets of highly predictive loci associated with sea age among populations and sexes within North America, indicating polygenicity and low rates of genomic parallelism. Despite low genome‐wide parallelism, we uncovered a set of conserved molecular pathways associated with sea age that were consistently enriched among comparisons, including calcium signaling, MapK signaling, focal adhesion, and phosphatidylinositol signaling. Together, our results indicate parallelism of the molecular basis of sea age in North American Atlantic Salmon across large‐effect genes and molecular pathways despite population‐specific patterns of polygenicity. These findings reveal roles for both contingency and repeated adaptation at the molecular level in the evolution of life history variation.

## Graphical abstract

Complex traits often exhibit complex underlying genetic architectures, and studies have begun to identify both large‐effect loci and polygenic patterns underpinning adaptation. Sea age represents a significant life history trait in Atlantic Salmon (*Salmo salar*), the genetic basis of which has been studied extensively in European populations, with repeated identification of large‐effect loci. In a survey of North American rivers, we found significant associations at the gene and SNP level with a large‐effect locus (*vgll3*) previously identified in European populations, but found that this pattern varied based on both sex and geographic region. Contrasting low genome‐wide parallelism, we uncovered a set of conserved molecular pathways associated with sea age that were consistently enriched among comparisons, indicating parallelism of the molecular basis of sea age in North American Atlantic Salmon across large‐effect genes and molecular pathways despite population‐specific patterns of polygenicity.

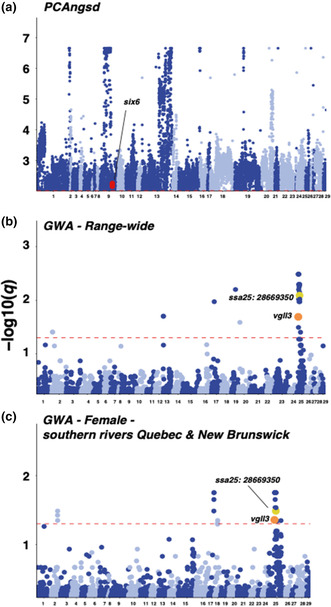

## 1 INTRODUCTION

A key component of understanding adaptive genetic variation is identifying the predictability of genomic patterns underlying repeated adaptation, thus providing insight into the possible molecular solutions for ecological challenges (Blount et al., 2018; Elmer & Meyer, 2011). Theoretical and empirical studies have indicated that variability among genetic architectures underlying adaptive traits may have consequences for their genomic parallelism (Bolnick et al., 2018; Yeaman et al., 2018). Parallelism at the genomic level is expected to occur with the greatest frequency in scenarios of shared ecological conditions and selection (Kaeuffer et al., 2012), phylogenetic similarity (Conte et al., 2012), shared standing variation (Ralph & Coop, 2015), and large‐effect loci controlling a large proportion of phenotypic variance (Yeaman, 2015). However, many traits exhibit polygenic architecture, and models of polygenic adaptation indicate small changes in allele frequency across many genetic pathways could reduce genomic parallelism (Barghi et al., 2019; Fagny & Austerlitz, 2021; Yeaman, 2015). Large‐scale follow‐up studies on the genetic basis of adaptive traits with previously identified large‐effect loci have also identified polygenic patterns explaining additional variation (Kreiner et al., 2021; Sinclair‐Waters et al., 2020), suggesting polygenicity may be common. Currently, the extent that simple and repeatable or polygenic and variable genetic architectures are more frequent during repeated adaptation remains unknown, requiring further study in the wild to characterize the genomic parallelism of adaptive traits.

Salmonids exhibit an extensive array of adaptive diversity (Klemetsen et al., 2003), with varying rates of underlying genomic parallelism (Jeffery et al., 2017; Salisbury et al., 2022). Atlantic Salmon (*Salmo salar*) is a culturally, ecologically, and economically significant species with an anadromous life cycle consisting of fresh water residency followed by a period at sea with enhanced growth rates prior to sexual maturation upon returning to fresh water to spawn (reviewed in Mobley et al., 2021). Sea age at first maturation varies both among individuals (Jonsson & Jonsson, 2007) and rivers (Hutchings & Jones, 1998) representing a variable life history strategy based on investment in reproduction timing and fecundity (Garant et al., 2003). This trait shows both genetic (Johnston et al., 2014) and environmental underpinnings (Friedland et al., 2000). Age at first maturation is demarcated by the number of winters spent at sea, also known as “sea age” or “sea winters”, with fish maturing after a single sea winter commonly known as 1SW or one‐sea‐winter salmon (1SW; Hutchings & Jones, 1998; Klemetsen et al., 2003) and fish first maturing after two or more winters at sea referred to as multi‐sea‐winter (MSW) salmon. Declines in Atlantic Salmon stocks across both Europe and North America (Lehnert, Kess, et al., 2019; Olmos et al., 2020), and reductions in the number of MSW salmon across years (Olmos et al., 2020) indicate recent selection against older age at first maturation (Czorlich et al., 2018) and highlight the need for greater understanding of this trait to inform conservation of life history variation within this species.

The genetic basis of sea age in Atlantic Salmon has been studied primarily in European populations (Barson et al., 2015; Sinclair‐Waters et al., 2022). Genomic investigation of this trait in rivers in Norway identified >30% of sea age variation was controlled by a large‐effect locus (*vgll3*, Barson et al., 2015) also associated with adiposity regulation and puberty onset in humans (Cousminer et al., 2013; Perry et al., 2014). However, significant associations with major effect loci in North American populations have varied among studies, indicating a complex genetic architecture (Boulding et al., 2019; Kusche et al., 2017; Mohamed et al., 2019).

Evolutionary history may play a role in explaining the discrepancy in identified genetic architectures underlying sea age between Europe and North America. Limited gene flow and trans‐Atlantic divergence may limit the genomic repeatability of this trait; differentiation between European and North American Atlantic Salmon populations is high (*F*_ST_ = 0.26) and genome‐wide (Lehnert et al., 2020), reflecting extensive periods of isolation (~600,000 years) for the potential evolution of distinct genetic architectures of maturation. However, secondary contact between North American and European lineages during deglaciation has also been identified (Rougemont & Bernatchez, 2018). Adaptive introgression of structural variants has been found in Newfoundland and Labrador (Lehnert, Bentzen, et al., 2019), suggesting potential for a shared genetic basis of maturation in regions with retained European variation.

Variation in regional ecological factors has also been shown to play a role in the expression of different sea age phenotypes, and the interaction between local conditions and genetic variation may shape the architecture of this trait across different locations. At a broad spatial scale, sea age in North American populations exhibits a strong latitudinal gradient and shows associations with precipitation, and river discharge (Hutchings & Jones, 1998; Power, 1981). Both river size and access to lacustrine habitat have also been shown to impact sea age, which may intersect with predicted trade‐offs between growth, sexual maturation, and survival (Klemetsen et al., 2003). North American populations exhibiting greater sea ages that are comparable to Norwegian populations are found at lower latitudes, primarily in the Maritimes and Gulf of St. Lawrence, whereas Newfoundland and Labrador populations exhibit low rates of MSW fish (Hutchings & Jones, 1998). Analyses of 1SW proportions across Norwegian populations have also uncovered a role for marine temperature in shaping this trait across rivers, together indicating variation across both marine and freshwater environments contributes to maturation age (Vollset et al., 2022).

Genetic analyses of sea age across local environmental conditions also indicate variation in how this trait is controlled across environments. Studies in differing light and feed availability conditions experienced in aquaculture environments (Ayllon et al., 2019; Mohamed et al., 2019), temperature exposures (Åsheim et al., 2023), and from different source populations have led to different detected genetic architectures (Boulding et al., 2019). This variation in trait expression has also corresponded with different detected associations at large effect loci (*vgll3*, *six6*) in a Norwegian population over temporal comparisons, indicating that these loci may be significant contributors to sea age only under specific conditions (Besnier et al., 2023). Together, these findings reveal complex interactions at regional and individual scales with environmental conditions can contribute to both genetic and plastic determination of sea age, implicating different genes in different contexts. However, genome‐scale investigations into sea age variation in wild Atlantic Salmon populations across North America have not yet been carried out and have the potential to reveal the genetic architecture and parallelism of this important life history trait.

Here, we explore two remaining questions about the genetic architecture of sea age in Atlantic Salmon: (1) is there evidence of genomic parallelism associated with sea age variation at the population and individual level in the Northwest Atlantic, and (2) is there detectable genomic evidence of polygenic adaptation associated with sea age in Atlantic Salmon. We used low‐coverage whole‐genome resequencing (WGS, *n* = 582) and a single‐nucleotide polymorphism (SNP) array (*n* = 658) to conduct population structure inference, genome‐wide association (GWA), and genome scans for variation associated with sea age in Atlantic Salmon sampled from 29 (26 SNP array, 8 WGS) rivers across eastern North America. To detect polygenic patterns associated with sea age, we then used machine learning to predict individual sea age from a panel of genome‐wide markers. Finally, we used gene‐set enrichment to detect biological and molecular function exhibiting polygenic selection associated with sea age. Our findings here provide insight into factors driving parallelism of individual genes and higher‐level conserved pathways underlying traits with variable and polygenic backgrounds.

## 2 METHODS

### 2.1 Sampling and genotyping: SNP array

**FIGURE 1:**
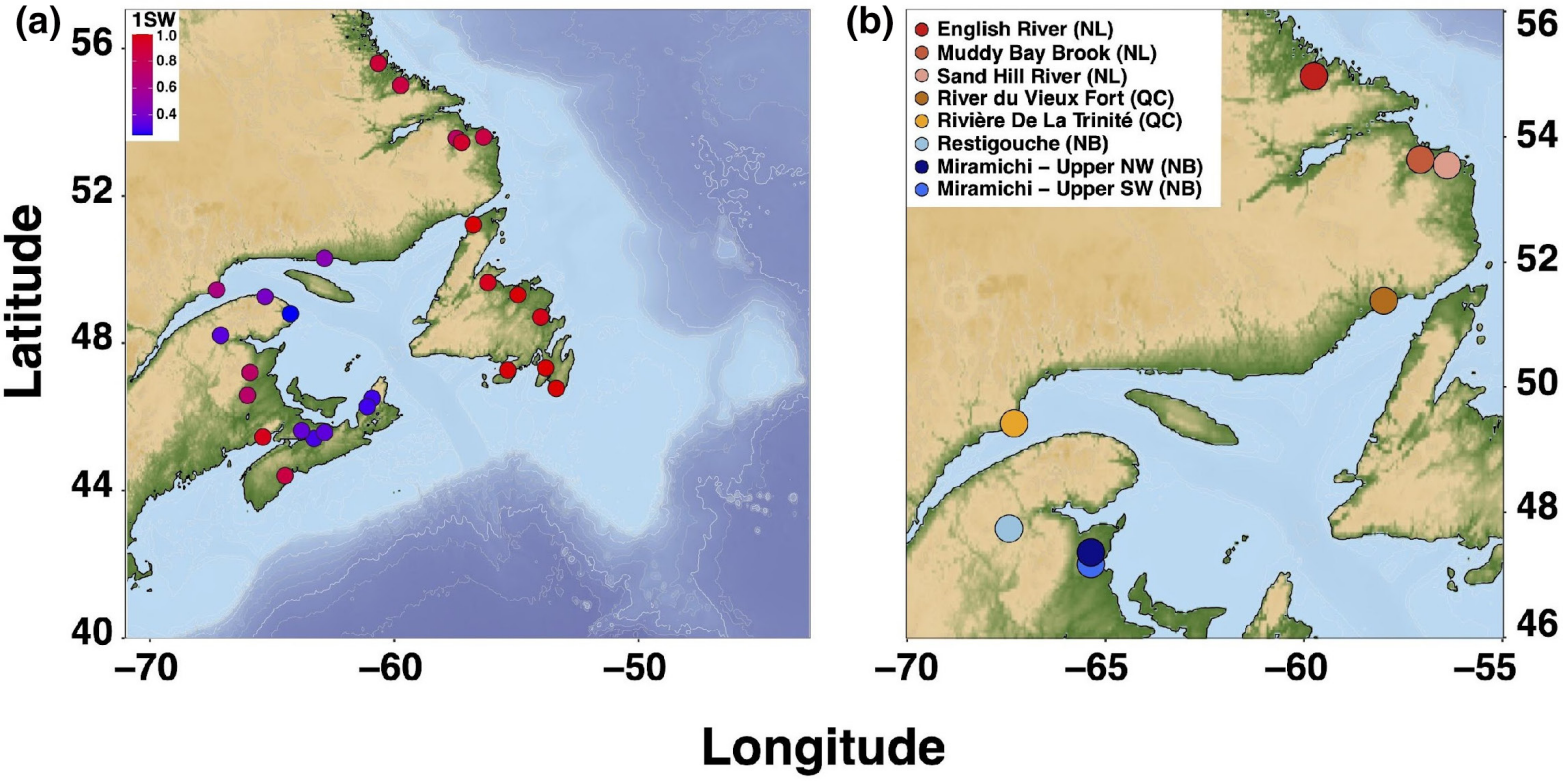
Map locations colored by 1SW proportion from 26 sampled rivers for individuals genotyped on the SNP array (a), and locations from eight sampled rivers for samples with individual sea age genotyped using whole‐genome resequencing (WGS) (b).

We first explored the relationship between genomic variation and sea age at the population‐level range‐wide acrossNorth American rivers. For range‐wide genomic analysis of population structure and sea age association, we used previously genotyped samples from published sources (combined from Lehnert et al., 2020; Wringe et al., 2018, full dataset described in Nugent et al., 2023), using a previously developed Atlantic Salmon 220K Axiom SNP array (Barson et al., 2015). We used genotype data at 97,566 SNPs that passed Axiom quality filters, with minor allele frequencies (maf) greater than 0.05. For all analyses, genotype data were retained for 658 individuals from 26 rivers of varying watershed size with population‐level mean proportion of maiden 1SW fish summarized from Canadian federal (Fisheries and Oceans Canada) and Quebec provincial (Ministère des Forêts, de la Faune et des Parcs) Atlantic Salmon counts from 1984 to 2020 (Figure 1a; Table 1). Individual genotypes were exported in plink format (Chang et al., 2015), and subsequent data filtration and conversions for analysis of SNP Array data were carried out in *plink* 1.90b6.16.

**TABLE 1:** Sampled river names, locations, collection years, life stages, timeframe during which sea age data were collected, proportion 1SW, and number of sampled individuals for rivers genotyped on the SNP array.

River	Latitude	Longitude	Region	Year collected	Life stage	Sea age data time period	Proportion 1SW	Sampled
Corneille	50.28	−62.88	Gulf St. Lawrence and Quebec	2018	Adult	1985–1988	0.462	28
Madeleine	49.23	−65.32	Gulf St. Lawrence and Quebec	2018	Adult	1984–2020	0.409	28
Matapedia	48.18	−67.142	Gulf St. Lawrence and Quebec	2018	Adult	1984–2020	0.334	15
Miramichi—Northwest	47.17	−65.94	Gulf St. Lawrence and Quebec	2016	Parr	1993–2013	0.733	24
Miramichi—Southwest	46.55	−66.04	Gulf St. Lawrence and Quebec	2016	Parr	1993–2013	0.719	23
Northeast Margaree	46.47	−60.919	Gulf St. Lawrence and Quebec	2018	Adult	1987–1996	0.289	12
Riviere de la Trinite	49.42	−67.305	Gulf St. Lawrence and Quebec	2012	Adult	1984–2019	0.634	49
Saint‐Jean (Gaspesie)	48.81	−64.43	Gulf St. Lawrence and Quebec	2018	Adult	1984–2019	0.307	28
Southwest Margaree	46.24	−61.122	Gulf St. Lawrence and Quebec	2018	Adult	1987–1996	0.289	14
Eagle River	53.53	−57.467	Labrador	n/a	n/a	2000–2007, 2016	0.747	21
English River	54.97	−59.75	Labrador	2010	Parr	2000–2019	0.859	27
Hunt River	55.57	−60.67	Labrador	n/a	n/a	2000–2007	0.893	19
Paradise River	53.42	−57.25	Labrador	2011	Parr	2001–2005, 2007–2020	0.924	19
Big Salmon	45.42	−65.41	Maritimes	2014	n/a	2000–2019	0.974	22
East River Pictou	45.54	−62.877	Maritimes	2018	Parr	1987–1996	0.34	23
LaHave	44.37	−64.5	Maritimes	n/a	n/a	1979–2010	0.853	22
North River NS	45.38	−63.31	Maritimes	n/a	n/a	1994–2019	0.299	22
River Philip	45.59	−63.82	Maritimes	2018	Parr	1987–1996	0.352	17
Sand Hill River	53.57	−56.35	Labrador	n/a	n/a	2002–2019	0.851	19
Campbellton	49.28	−54.934	Newfoundland	2009	Parr	2011–2020	0.995	25
Garnish	47.23	−55.35	Newfoundland	2009	Parr	2015–2020	0.992	22
Great Rattling Brook—Exploits	49.62	−56.17	Newfoundland	2010	Parr	2000–2002, 2011–2020	0.971	26
Northeast Brook Trepassey	46.74	−53.36	Newfoundland	2010	Parr	2000–2010, 2012	1	25
Northeast Placentia River	47.29	−53.796	Newfoundland	2017–2019	Parr	2000, 2015–2019	1	81
Terra Nova River	48.67	−54	Newfoundland	2009	Parr	2000–2001, 2005, 2011, 2014–2020	0.977	29
Western Arm Brook	51.19	−56.765	Newfoundland	2016	Adult	2000–2020	0.996	18

### 2.2 Sampling and genotyping: WGS

To identify associations between individual sea age and genomic variation, we carried out low‐coverage whole‐genome resequencing as in Therkildsen and Palumbi (2017) of 582 male and female wild salmon from eight rivers in North America (Figure 1b), spanning southern rivers in Quebec and New Brunswick, and northern rivers in Quebec and Labrador. To minimize impacts of population structure or sex on individual GWA results, these individuals were grouped into separate sets of MSW and 1SW fish of each sex from each sampling region (Table 2). Atlantic Salmon were caught at fish‐counting fence traps or by angling and were sampled for fin clips for DNA extraction and scales to identify sea age. Sex was confirmed for each individual with PCR of the *Salmo salar* sex determining region *sdY* from Yano et al., 2013 (SS sdY S: “GGCCTATGCATTTCTGATGTTGA”, SS sdY AS: “AGAGGATTGAACGGTCAGAGGAG”). To prepare whole‐genome resequencing libraries, we extracted genomic DNA following Mayjonade et al. (2016), using Longmire's buffer (Longmire et al., 1997) for tissue lysis. We then used a modified version of the protocol described in Therkildsen and Palumbi (2017) and scaled down reaction volumes of Nextera DNA Flex Library Prep Kits (Illumina) to 0.13× the volume of the standard Illumina protocol. For library amplification, we used Kapa Hi‐Fidelity Library Amplification Kits (Roche) in 20‐μL reactions with 4‐μL Nextera Unique Dual Indexes Set A (Illumina). We then quantified libraries by Qubit (ThermoFisher) and checked average fragment size using an Agilent Bioanalyzer. We normalized libraries from 96‐well plates to equimolar concentrations prior to combining as a single pool per lane of sequencing. Libraries were then sequenced on six lanes of an Illumina NovaSeq6000 S4 at the Genome Quebec Centre d'Expertise et de Services.

**TABLE 2:** Sample size of each sea age class and sex, with sampling locations and years, for WGS dataset.

River	Region	Male—Single sea winter	Male—Multiple sea winters	Female—Single sea winter	Female—Multiple sea winters	Latitude	Longitude	Sampling years
	Total	155	116	140	171			
	Southern Rivers	69	79	58	82			
Miramichi Upper Northwest NB		21	17	28	17	47.17	−65.94	2018
Miramichi Upper Southwest NB		12	16	9	20	46.55	−66.04	2017–2018
Restigouche NB		16	32	1	25	47.99	−66.89	2019
Riviere de la Trinite QC		20	14	20	20	49.42	−67.3	2011–2017
	Northern rivers	86	37	82	89			
English River NL		23	9	21	26	54.97	−59.75	2018–2019
Muddy Bay Brook NL		20	0	19	20	53.638	−57.0651	2016–2019
Sand Hill River NL		23	20	22	23	53.548	−56.393	2018–2019
River du Vieux Fort QC		20	8	20	20	51.38	−57.99	2011, 2015–2017

Quality of sequenced libraries was checked using FastQC, (Andrews, 2010). We used *cutadapt* 2.1 (Martin, 2011) to remove the leading 15 bases, adapter content, bases falling below of q score of 10, and any read with less than 40 remaining base pairs. Trimmed reads from each individual were aligned to the 29 chromosomal contigs from the ICSASG_V2 *Salmo salar* reference assembly (GCF_000233375.1) using *bwa mem* 0.7.17 (Li, 2013). Subsequent steps followed best practice recommendations for *Genomic Analysis Toolkit* (GATK 3.7, DePristo et al., 2011): We first removed duplicate reads using the *PicardTools* 2.20.6 *MarkDuplicates* function. Deduplicated reads were then sorted and realigned around potential insertions and deletions using *RealignerTargetCreator* and *IndelRealigner* functions in *GATK*. We estimated alignment depths on realigned .bam files using *mosdepth* 0.3.3 (Pedersen & Quinlan, 2018), and alignment rate and read numbers using *samtools flagstat*. This processing resulted in a set of deduplicated, realigned reads consisting of a mean of 54,538,983 paired reads per individual (median 56,011,607, SD = 13,145,588) with a mean 97.59% alignment rate (median = 97.59%, SD = 0.14%) on an average sequencing depth of 3.23×, (median = 3.31×, SD = 0.782).

Next, we estimated genotype likelihoods, as well as directly called genotypes, for each individual using Analysis of Next‐Generation Sequencing Data (*ANGSD* 0.935, Korneliussen et al., 2014). We output genotype likelihoods for each chromosome as beagle format likelihood files (Browning & Browning, 2007) for all SNPs passing quality filters (‐*minMapQ* 30 ‐*minQ* 20 *‐SNP_pval* 2e‐6 *‐uniqueOnly* 1 *‐remove_bads* 1) and ensuring greater than 80% (‐*minInd* 470) of individuals had genotypes, and a minimum of 500 reads were present per locus (*‐setMinDepth* 500). Genotype calls from each chromosome were exported as bcf files using the ‐*dobcf* flag and converted to vcf files (Danecek et al., 2011) using *bcftools* 1.11 (Li et al., 2009). We then performed phasing and imputation of vcf files using *Beagle* 4.0 (Browning & Browning, 2007) resulting in 9,895,443 SNPs. To test the accuracy of imputed genotypes, we estimated allele frequency for the dataset using both the ‐*freq* function in *vcftools* 0.1.16 (Danecek et al., 2011) on imputed genotypes and the *‐domaf* function on realigned, deduplicated bam files in ANGSD.

### 2.3 Population structure

To account for potential covariation in ancestry and sea age in GWA, we first estimated population structure of SNP array data using principal component analysis (PCA) in the R package *pcadapt* (Privé et al., 2020). For retaining relevant PC axes for GWA and outlier detection, we followed Cattell's methodology (1966) and retained PCs at break points in the amount of variation explained per axis, as suggested for *pcadapt* (Luu et al., 2017). Using this approach, we used scree plot visualization to identify *K* = 5 ancestral populations (Figure S1). We also estimated population structure using the sparse non‐negative matrix factorization algorithm (*snmf*, Frichot et al., 2014) implemented in the *R* package *LEA* (Frichot & Francois, 2015). This approach models ancestral populations from which contemporary genomes are derived based on observed allele frequencies. We selected *K* = 5, based on PCA results, and inspection of cross‐entropy criteria which showed highest rates of reduction in cross‐entropy until *K* = 5 (Figure S1). However, we acknowledge additional changes in percent variation explained and cross‐entropy values past *K* = 5, and alternative values of *K* are likely also valid using this approach (Lawson et al., 2018) especially given known hierarchical population structure in Atlantic Salmon (Bradbury et al., 2014; Lehnert et al., 2023). We focus on these first five primary ancestry sources, as our goal here is to characterize the main potential sources of broad‐scale population structure that may be associated with 1SW proportion.

We directly tested for covariation of 1SW proportion and population structure using variance partitioning (Capblancq & Forester, 2021; Legendre & Legendre, 2012) with the *varpart* function in the *vegan* R package (Oksanen et al., 2020). Using this function, we tested the extent that variation in 1SW proportion can be explained by the first five principal components of genetic variation from PCA. Significance of the variance partitioning model was explored by predicting 1SW proportion from genetic PCs in a redundancy analysis (RDA) model with the *rda* function, and the significance of this model was tested with the *anova.cca* function with 999 permutations.

Population structure in WGS samples was quantified using genotype likelihoods in *PCAngsd* 1.02 (Meisner & Albrechtsen, 2018). This method accommodates low‐coverage whole‐genome resequencing data through modeling genotype uncertainty in PCA for continuous estimation of population structure and in non‐negative matrix factorization for estimation of admixture components. Selection of the optimal number of principal components was handled here internally in *PCANGSD* using the minimum average partial test. Selection of the *K* number of ancestral populations was also carried out in *PCANGSD* based on likelihood convergence. Details of these methods can be found in Meisner and Albrechtsen (2018). As with the SNP array data, we also used variance partitioning to directly estimate the proportion and significance of variation in principal components of genetic structure that explained individual sea age, using the first two PCs from WGS data with all samples included.

### 2.4 PCA selection scan

We identified the genome‐wide landscape of differentiation associated with population structure by carrying out genome scans with per‐SNP significance estimates from PCA loadings. This approach leverages PCA to identify separate PC axes associated with different sources of population structure. Genomic regions that show strong associations with these axes are considered potential targets of divergent selection between populations based on allele frequency differences between projected clusters in ordination space (Duforet‐Frebourg et al., 2016). Loci exhibiting signatures of selection associated with population structure were detected in SNP array data by estimating *p*‐values for each SNP using the Mahalanobis method in *pcadapt* with *K* = 5. This method measures the strength of association of SNPs with *K* retained principal components; outliers will exhibit stronger associations with PC axes relative to the majority of loci genome‐wide (Luu et al., 2017). We adjusted *p*‐values estimated from *pcadapt* for false discovery rate (FDR, Storey & Tibshirani, 2003) using the *qvalue* R packages (Storey et al., 2015) and selected SNPs with *q* < 0.05 as exhibiting significant signatures of selection associated with population structure. This threshold has recently been identified to provide an acceptable trade‐off between false positive inclusion and failure to detect relevant loci due to test stringency (Chen et al., 2021).

To detect regions of elevated population structure associated with divergence in WGS data, we estimated *p‐*values for each SNP from its loading on each PC axis estimated in *PCANGSD* (Meisner et al., 2021), assuming a χ^2^ distribution of PC loadings, and then selected FDR‐adjusted *q* values <0.05.

### 2.5 Genome‐wide association with sea age

**FIGURE 2:**
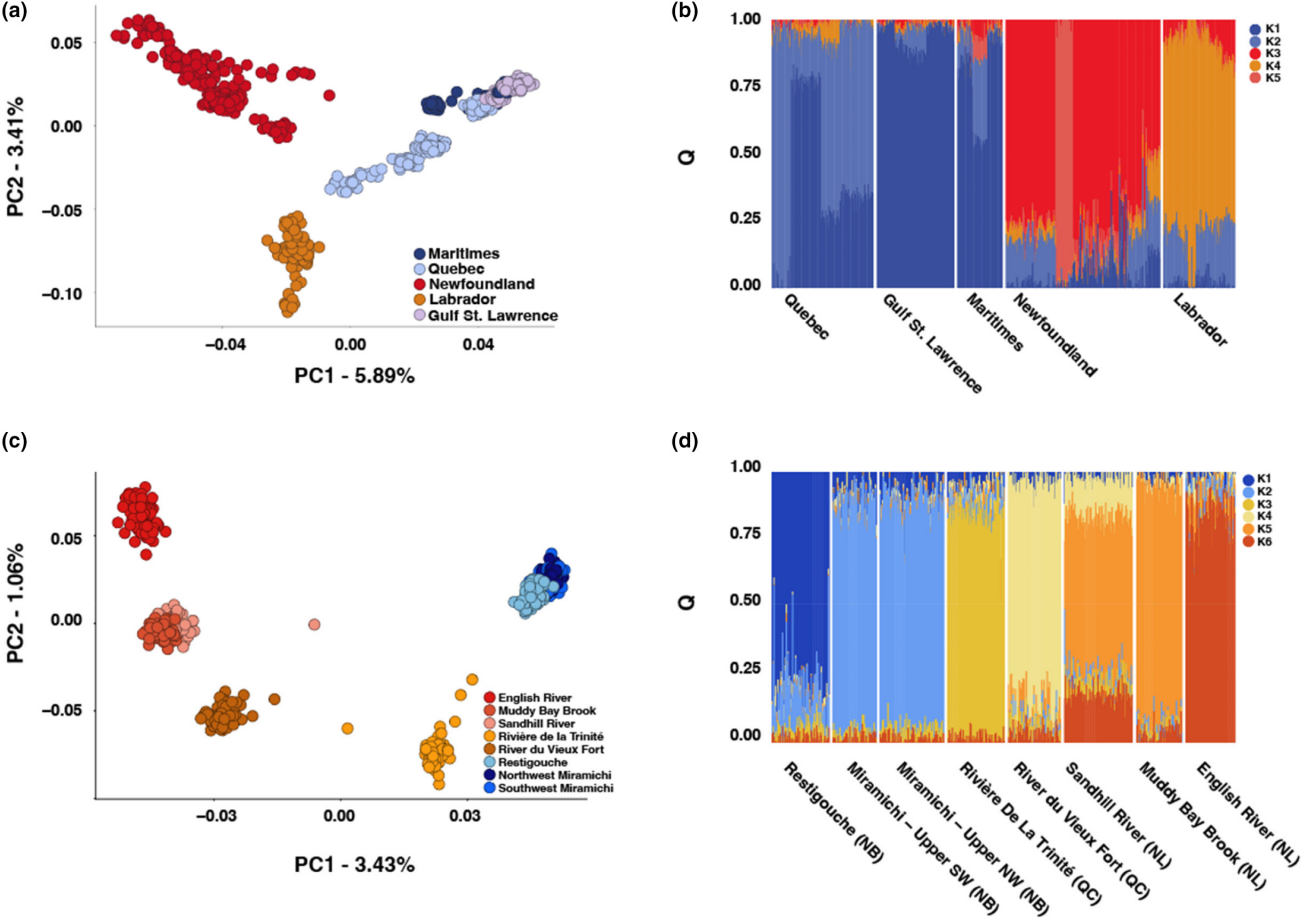
Population structure inferred from principal component (PC) analysis in *pcadapt* colored by geographic origin (a), individual admixture proportions identified from sparse non‐negative matrix factorization in *LEA* of individuals genotyped on the SNP array (b), population structure inferred from principal component analysis (c), and admixture proportions across regions identified from non‐negative matrix factorization (d) in *PCANGSD* using whole‐genome resequencing (WGS) samples.

To explore the association between range‐wide genetic and sea age variation, we conducted genome‐wide association with SNP array data using population‐level 1SW proportions, which reflect the average number of 1SW fish per population. Per‐SNP associations with 1SW proportion were estimated using latent factor mixed models with *LFMM 2* in *lfmm* (Caye et al., 2019), using the “gif” parameter to correct for genomic inflation driven by potential systematic biases in *p*‐values. We specified *K* = 1 to test for association without correction for population structure and *K* = 5 to account for variation associated with the retained PC axes which separated individuals by river and 1SW proportion (Figure 2b). To characterize multilocus associations with 1SW proportion, we also conducted RDA with the *rda* function in *vegan*, using 1SW proportion to predict genome‐wide variation, and selected the top 1% of SNPs based on absolute value of per‐SNP RDA scores.

For WGS data with individual phenotypes, we used the association functions in *ANGSD* to test for genomic association with individual sea ages from genotype likelihoods using a latent genotype model (‐*doAsso* 4, Jørsboe & Albrechtsen, 2022). Individual sea age was included as a binary phenotype, as all samples compared in this study exhibited a sea age at first maturity of 1 or 2 years, and only a small subset (*n* = 11) exhibited total sea age greater than two. These individuals were assigned an initial sea age of 1 years, indicating repeat spawning of 1SW fish rather than MSW maturation. Association analysis was carried out on genotype likelihoods from the phased and imputed vcf of all samples, without correction for population structure, as well as with the scores from the first two PCs as covariates. To identify the genetic architecture of this trait at finer spatial scales, we then repeated this analysis within each regional grouping (within southern rivers in Quebec and New Brunswick, and within northern rivers in Quebec and Labrador) and sex, with the first PC included in each analysis as a covariate to correct for population structure. For region‐level GWA, we re‐estimated PC scores per individual within each region separately (North and South). For all GWA with WGS data, we estimated the lambda inflation factor (Devlin & Roeder, 1999) as evidence of population structure‐driven confounding using the *P_lamba* function in the QCEWAS R package (Van der Most et al., 2017). As a second measure of genomic divergence associated with sea age, per‐locus Weir & Cockherham's *F*_ST_ (Weir & Cockerham, 1984) was estimated with *vcftools* from phased and imputed vcf files between these same groups based on separation by sea age class.

### 2.6 Machine learning prediction of sea age from genotype dosage

As many genomic regions that fall below genome‐wide statistical significance may still carry substantial predictive information (McGaugh et al., 2021), we next tested the utility of a machine learning approach to predict individual sea age using WGS data. We first selected the top 100, 200, 300, 400, and 500 loci with the highest likelihood ratio test score from each population structure‐corrected GWA using WGS data to use as predictors in a random forest model (Breiman, 2001), as in Hess et al. (2016) and suggested in Brieuc et al. (2018) to minimize overfitting. We then used continuous genotype dosages, which allow for quantifying the uncertainty at individual loci from differences in read depth or SNP quality for each locus (Zheng et al., 2011), as predictors of trait variation. Prediction of sea age for each sex and regional grouping was carried out using a random forest classification model (Breiman, 2001) in the *randomForest* R package (Liaw & Wiener, 2002), setting 25,000 trees and 80% of SNP sample size for the *mtry* parameter for each analysis. To estimate the prediction accuracy of each model, we estimated out‐of‐bag error (OOB), which quantifies the number of misidentified samples from a testing dataset with known categories. For each tree, we specified a holdout of half of individuals from each sea age class, equalized by sample size, for testing and training, similar to the approach used by Brieuc et al. (2018). We then compared the accuracy of the selected panel of SNPs by generating panels of 100–500 randomly selected SNPs and calculated OOB for these panels to quantify classification error of random SNPs relative to those with elevated GWA significance. The trade‐off between sensitivity and specificity was estimated for the full model of all samples for 500 SNP prediction with a receiver operator characteristic (ROC) curve using the R package *pROC*.

### 2.7 Gene‐set enrichment

To identify signals of polygenic selection at the pathway level, we next used the gene‐set enrichment approach implemented in the *polysel* set of R tools (Daub et al., 2013), which tests for shared patterns of elevated values of a population‐genetic parameter among genes within pathways. This method differs from gene ontology enrichment methods by testing for elevation of parameters across all genes within a pathway as evidence of polygenic selection, whereas gene ontology enrichment relies on greater than expected similarity of function in a set of preidentified outlier genes. We downloaded gene information for Atlantic Salmon from http://www.ncbi.nlm.nih.gov/gene and KEGG pathway information (Ogata et al., 1998) for Atlantic Salmon from https://www.ncbi.nlm.nih.gov/biosystems/. We then selected the highest scoring SNP of each tested statistic for each gene and ran gene‐set enrichment on: Likelihood ratio test scores from genome‐wide association with sea age in the WGS datasets, per‐SNP PC1 scores from PCAngsd, −log10(*p*) *LFMM* scores from SNP array association with 1SW proportion at *K* = 1 and *K* = 5, and PC1 scores from *pcadapt*. We set a minimum of five genes per set, resulting in 237 sets and 11,437 genes analyzed for whole‐genome data, and 209 sets and 5507 genes analyzed for Axiom Array data. Significantly enriched KEGG pathways were identified using a *q* threshold of 0.05.

## 3 RESULTS

### 3.1 Population structure

Population structure analyses using the SNP array uncovered structuring associated with regional differences in North American Atlantic Salmon populations (or 1SW salmon; Figure 2a,b). We found that the first two PCs separated populations partially by regional differences in 1SW proportion among sampled rivers (primarily along the first PC axis, Figure S3), explaining most of the genetic variation, after which percent variation explained fell below 2% (Figure S1). Admixture analysis using *snmf* also supported multiple genetic clusters within broader geographic regions (Figure 1b). Variance partitioning of 1SW proportion by principal component scores of genetic variation identified a significant (*p* < .001) and large proportion of variation explained by PC1 (61%), with smaller but significant contributions from PC2 (1%) and PC5 (4%).

Consistent with our experimental design, we did not find a large proportion of genome‐wide population structure associated with individual age classes of WGS individuals, in contrast to population‐genetic clustering by river‐level 1SW proportion observed with the SNP array. Population structure inferred using genotype likelihoods in *PCAngsd* among the eight rivers in the whole‐genome resequencing dataset revealed regional separation between southern and northern rivers, and further clustering at the river level (Figure 2c). *PCAngsd* identified *K* = 6 as optimally describing population structure (Figure 2d) and revealed more similar ancestry among close rivers within the same region. Variance partitioning of sea age variation by PCs revealed a small but significant proportion of variance in individual sea age explained by PC1 (1.7%, *p* < .001), indicating reduced influence of population structure on individual‐level GWA statistics compared to SNP array data.

### 3.2 PCA‐based signatures of selection

**FIGURE 3:**
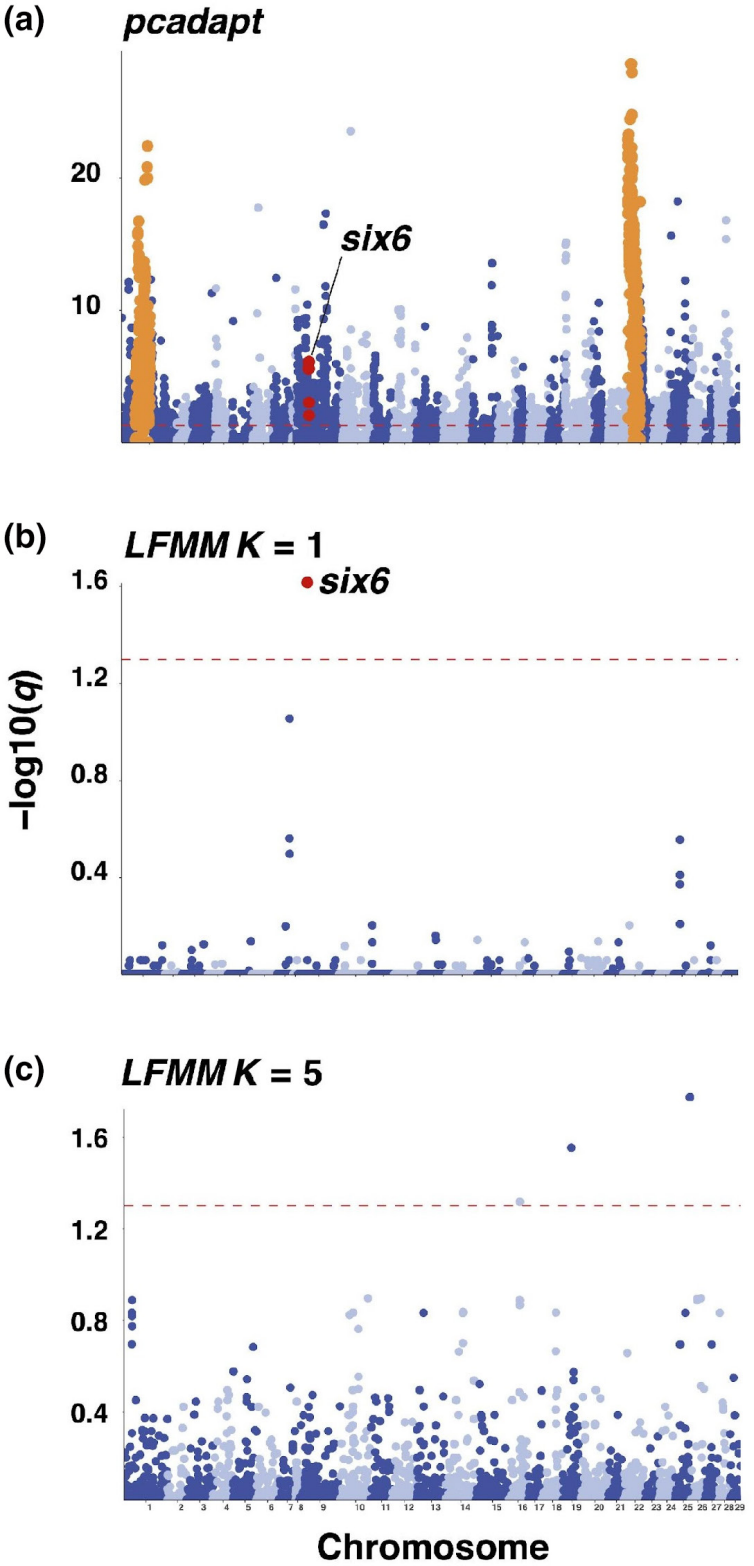
Genome‐wide distribution of Axiom array SNPs exhibiting significant association (red dotted line, *q* < 0.05) with population structure in *pcadapt* (a), 1SW proportion in river of origin in LFMM with *K* = 1 (b) with red circles indicating the location of SNPs within *six6*, and orange circles indicating location of karyotypic variants identified by  Lehnert, Bentzen, et al. (2019).

Using PCA‐based scans for selection associated with population structure among populations genotyped on the SNP array, we uncovered many genomic regions exhibiting elevated divergence, including a previous sea age‐associated locus, *six6* (Figure 3a). We identified 3593 SNPs with elevated significance in PCA using *pcadapt* (*q* < 0.05), distributed genome‐wide. These outliers overlapped 1139 genes (Table S1), as well as the introgressed European karyotypic variant regions (Lehnert, Bentzen, et al., 2019) on chromosomes ssa01and ssa23 (*n* = 559).

**FIGURE 4:**
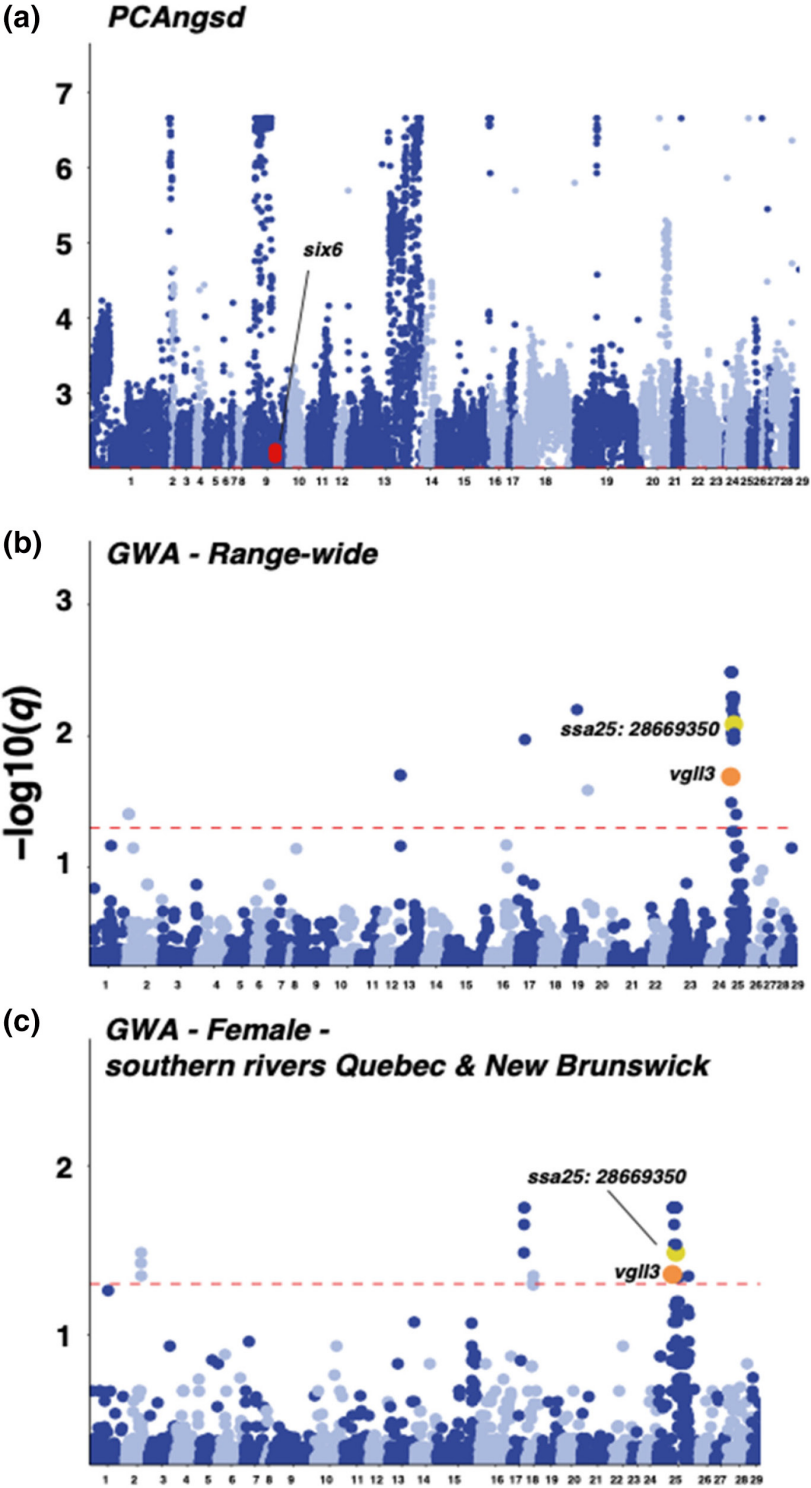
Genome‐wide distribution of outlier SNPs identified from low‐coverage whole‐genome resequencing (WGS) exhibiting significant association (red dotted line, *q* < 0.05) with population structure in *PCAngsd* (a), the top 5000 most significant SNPs associated with individual sea age across all samples (b) individual sea age within female fish sampled in southern rivers in Quebec and New Brunswick Rivers (c), with red circles indicating the location of SNPs within *six6*, orange indicating within *vgll3*, and yellow indicating the location of the SNP with highest explanatory power for sea age on *ssa25* identified by Sinclair‐Waters et al. (2022).

Selection scans of WGS samples using *PCAngsd* identified 52,986 significant SNPs overlapping 3262 genes (Table S2), again including *six6* (Figure 4a). Allele frequencies at the SNPs with the highest PCA loading differed substantially between North and South datasets but did not show divergence between sea age classes (Figure S4). Significant association of population structure with karyotype variants was negligible (*n* = 18, ssa01, *n* = 1 ssa23) among WGS individuals.

### 3.3 Genome‐wide association with sea age

Using SNP array data and 1SW proportions from 26 rivers across North America, we found 11 associated SNPs at *K* = 1 and one overlapping gene: *six6* (Figure 3b; Table S3). This pattern of association at six6 was also strongest among the 976 SNPs identified used RDA, overlapping 312 distinct genes (Table S4), and we found complete overlap of the 11 detected SNPs with *K* = 1 and RDA and *pcadapt* outliers. However, after controlling for population structure in *LFMM* using *K* = 5, we did not detect an association with *six6* and instead identified six significantly associated SNPs overlapping two genes, with the strongest signal of association found at a tissue factor pathway inhibitor‐like gene (Table S3). We found both sets of SNPs detected with *LFMM* exhibited complete overlap with RDA outliers, and high overlap with *pcadapt* (*K*1 = 11, 100%, *K*5 = 3, 50%) outliers.

Genome‐wide association using individual phenotypes using the WGS data instead identified 32 significant loci, and an overlap with *vgll3* as the only significantly associated gene in the total dataset analysis (Figure 4b; Table S5). The lambda inflation factor was 1.0635, consistent with that identified in Barson et al. (2015). In a finer‐scale analysis of female fish from southern rivers, we identified 35 significant SNPs, overlapping with three genes: a predicted glutamate receptor, NMDA 2B‐like on ssa02, a butyrophilin subfamily 2 member A1‐like gene on ssa18, and *vgll3* (Figure 4c; Table S5). In both comparisons, we uncovered a significant association at a SNP on ssa25 (Figure 4c; Figures S4 and S5) showing the strongest sea age association in European Atlantic salmon, recently identified by Sinclair‐Waters et al. (2022). No significant associations were identified within the other subsets at *q* < 0.05. In comparison with the genome‐wide average, we identified elevated *F*_ST_ values among the top 100 most significant SNPs, but no increased PC1 association (Table 3), indicating that SNPs identified in GWA show elevated differentiation between sea age classes, but do not show elevated differentiation associated with population structure. Comparing *F*_ST_ to uncorrected GWA LRT revealed high and significant correlation (*r* ^2^ = .914, *p* < 1 × 10^−15^), indicating imputation and genotype likelihood estimates captured similar allele frequency variation across the genome. Similarly, direct comparison of allele frequencies estimated in *ANGSD* from genotype likelihoods or *vcftools* from imputed genotypes showed strong correlation (*r* ^2^ = .986, *p* < 1 × 10^−15^).

**TABLE 3:** *F*_ST_ between 1SW and MSW, PC1 loading in PCANGSD for the top 100 associated SNPs and genome‐wide loci across separate GWA of sea age, with * denoting a significant difference in parameters between top SNP and genome‐wide SNP comparisons.

GWA group	*F*_ST_*		PC1 loading	
	Top 100 SNPs	Genome‐wide SNPs	Top 100 SNPs	Genome‐wide SNPs
Female—southern rivers Quebec and New Brunswick	0.161	0.0007	0.759	1.02
Male—southern rivers Quebec and New Brunswick	0.0928	0.0002	0.945	1.02
Female—northern rivers Quebec and Labrador	0.0854	0.00009	0.923	1.02
Male—northern rivers Quebec and Labrador	0.1533	0.0003	0.677	1.04

### 3.4 Machine learning prediction of sea age

**FIGURE 5:**
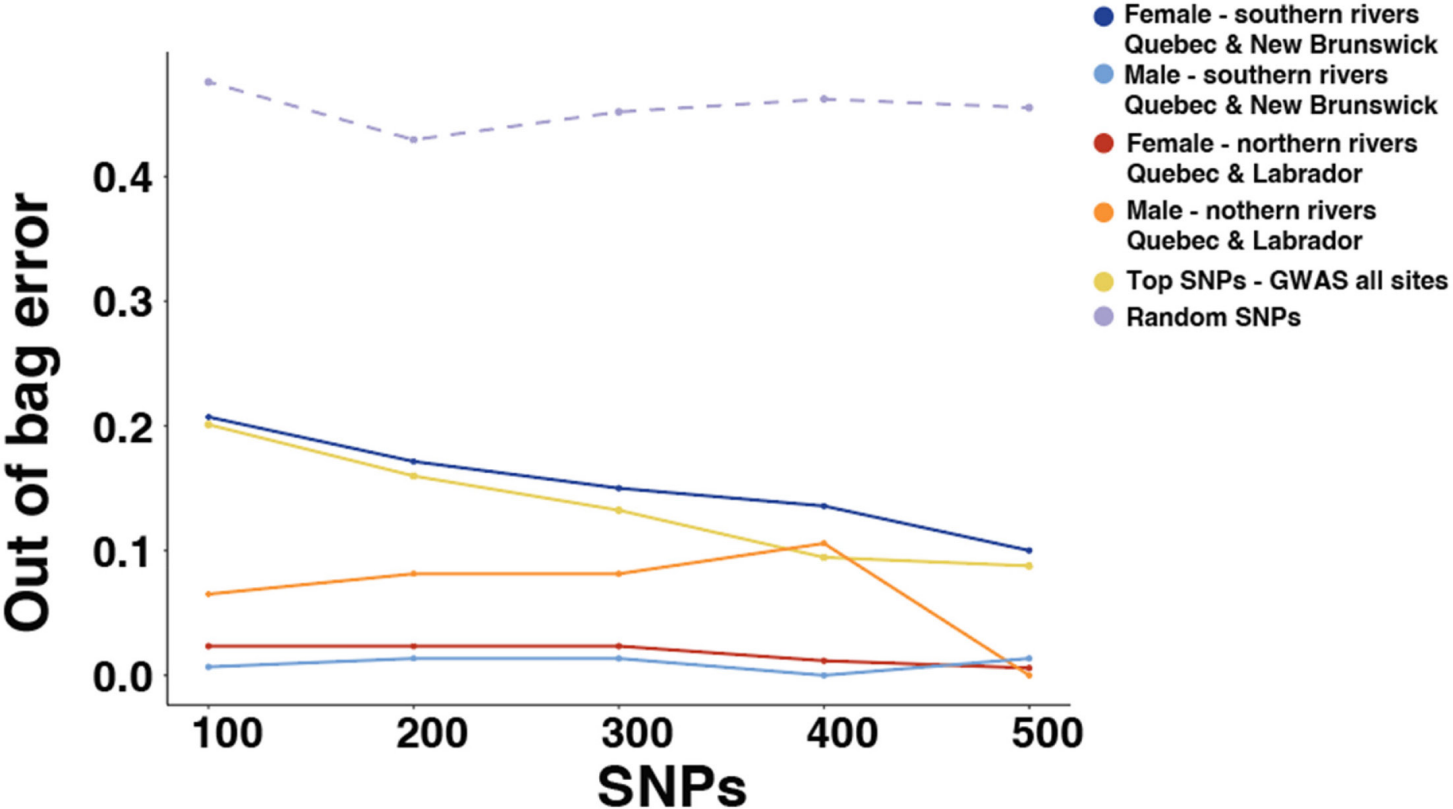
Random forest out‐of‐bag classification error rates across SNP panel sizes (100–500) for the highest ranking SNPs in GWAS for all samples stratified by sex and region from whole‐genome resequencing (WGS). Randomly selected SNPs are represented with a dashed line.

Across datasets, the 100 most significant SNPs based on LRT score could predict sea age with greater than 75% accuracy (Figure 5). Increasing the number of SNPs within each dataset increased accuracy to greater than 80% overall, and 90% in all but the female southern rivers dataset and total dataset models. Investigation of the ROC curve for the total dataset identified both high sensitivity and specificity of random forest models using 500 sea age‐associated SNPs, inferred from rapid rise above the diagonal slope of a random classifier (Figure S6). Across models with randomly selected SNPs, we found consistently low prediction accuracy (~45%), even with the 500 SNP panel, indicating reduced predictive capacity of comparably sized panels of randomly selected genome‐wide SNPs.

### 3.5 Gene‐set and gene ontology enrichment

**FIGURE 6:**
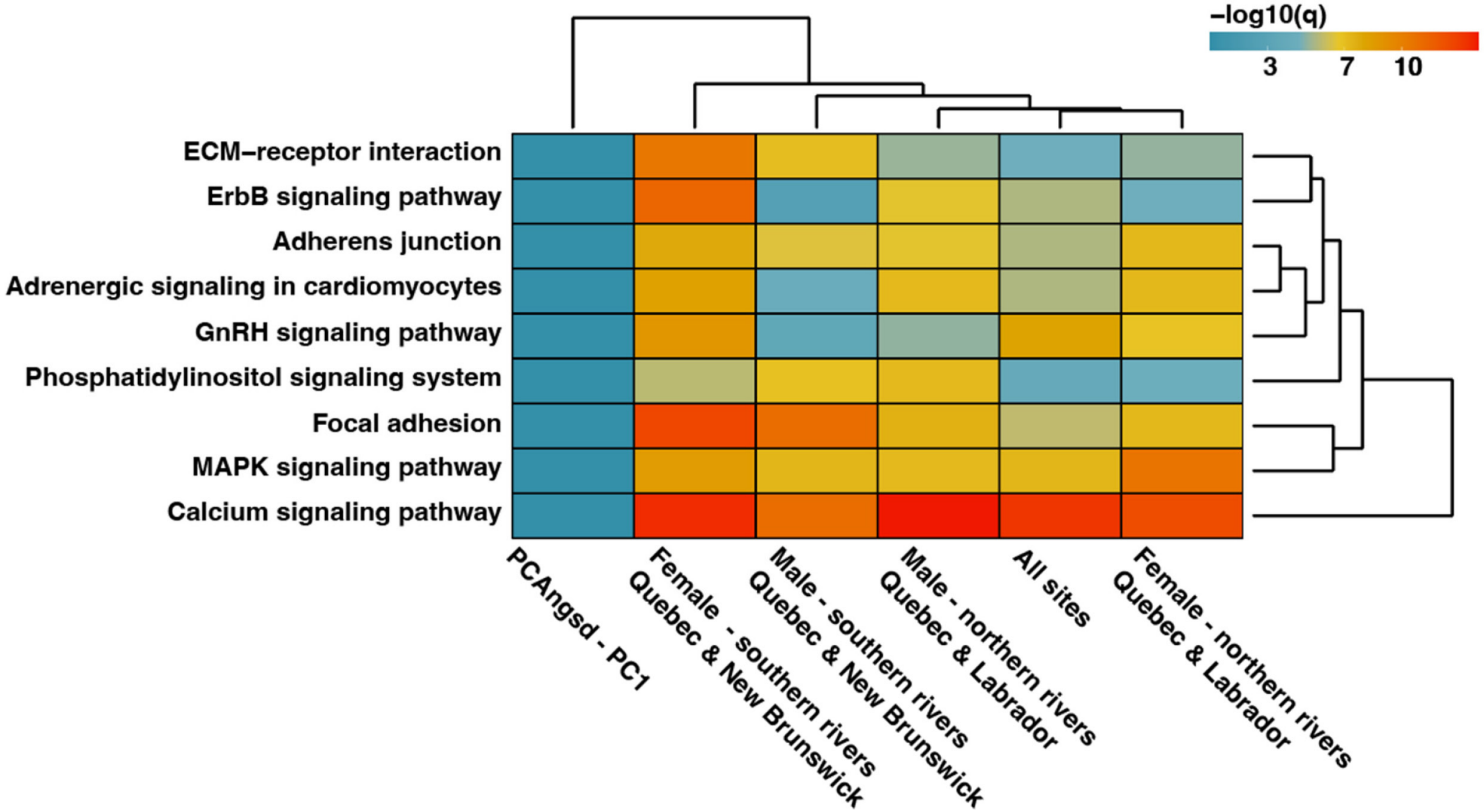
Hierarchical clustering of the nine parallel and highly significant (*q* < 0.001) KEGG pathways identified using gene‐set enrichment across groups using GWAS scores from whole‐genome resequencing (WGS) data, with gene‐set enrichment PCANGSD results for WGS data included for comparison.

Using gene‐set enrichment, we identified 37 significantly enriched KEGG pathways among GWA comparisons of individual sea age in the WGS data, many of which were significantly enriched among multiple comparisons (Figure 6; Table S6). In contrast, we did not identify significant enrichment among PC scores from WGS or SNP array analyses, or −log10(*p*) values from range‐wide SNP array *LFMM* analyses. Hierarchical clustering of −log10(*q*) values from gene‐set enrichment revealed a core set of nine processes corresponding to developmental, osmoregulatory, neurological, and cardiovascular functions that were most significantly enriched among all groups, such as the phosphatidylinositol signaling system, adrenergic signaling in cardiomyocytes, the GnRH signaling pathway, and focal adhesion. We found the most significant enrichment among all groups in the calcium signaling pathway (Figure 6).

## 4 DISCUSSION

Identifying the genomic basis of adaptive traits in wild populations is key to understanding how evolutionary processes give rise to diverse and ecologically important phenotypes (Bolnick et al., 2018). In Atlantic Salmon, evolutionary history, marine and freshwater ecological and habitat variability, and plasticity likely shape the genomic repeatability of sea age, but its genomic basis in North America has yet to be identified. Here, we characterized the genetic architecture of sea age variation in North American Atlantic Salmon using SNP array and WGS genomic datasets to test whether this genetic architecture is repeatable at two spatial scales: between European and North American populations, and within North American populations. Consistent with the expectation that large‐effect loci will exhibit higher rates of parallelism (Barghi et al., 2019; Yeaman, 2015), significant associations with sea age in North America overlapped with a large‐effect European sea age locus at the gene and SNP level (*vgll3*). Within North America, we also found polygenic adaptation within regions and sexes with no overlap of associated loci, whereas gene‐set enrichment revealed repeatable patterns of selection within conserved pathways associated with maturation. Our findings indicate contingency in the genetic architecture of sea age in Atlantic Salmon, but high parallelism of core molecular pathways and large‐effect loci in generating a diversity of at‐sea maturation strategies.

### 4.1 Detection of known maturation loci

Our results across association analyses in the WGS dataset revealed parallel genetic architecture of sea age variation between North American and European Atlantic Salmon at the previously identified large‐effect locus *vgll3*. Significant GWA results using WGS data paired with individual phenotypes and elevated differentiation (*F*_ST_) indicated strong selection at *vgll3* in female fish in southern rivers in Quebec and New Brunswick. This finding is concordant with past research in European populations, both in identifying strong selection and sea age association at *vgll3*, and in finding sex‐specific patterns of association and dominance at *vgll3* (Barson et al., 2015). Similar patterns of association with large‐effect loci underlying sex‐specific maturation and migration have also been revealed across the broader salmonid lineage, indicating large‐effect loci may be common in genetic architectures for distinct life history strategies (Pearse et al., 2019; Thompson et al., 2020).

Additionally, we uncovered a SNP‐level pattern of parallelism at a *vgll3*‐adjacent region between European and North American Atlantic Salmon. This region has previously been identified as highly significant in explaining sea age variation in European Atlantic Salmon in a recent candidate gene study (Sinclair‐Waters et al., 2022). Our observation is consistent with the expectation that large‐effect loci are more likely to exhibit parallelism (Yeaman, 2015). The source of this parallelism, either through ancestral variation, repeated evolution, or migration, remains unknown and is a clear goal for follow‐up studies (Lee & Coop, 2017). However, our results suggest that associations at *vgll3* and the *vgll3*‐adjacent SNP varied across rivers and sexes, indicating heterogeneity in the genetic architecture of maturation across the North American range despite gene and SNP‐level genetic parallelism. This finding is consistent with a past amplicon‐based study (Kusche et al., 2017), which similarly identified significant variation in associations of *vgll3* and sea age dependent on sampled river. Variation in associations with *vgll3* and sea age across North American rivers observed by Kusche et al., 2017 was attributed to a potential low proportion of MSW fish with advanced sea age (>3SW) in amplicon‐genotyped samples. Similarly, our sampling of fish across eight North American locations identified predominantly 2SW fish and the small subset of fish with age greater than 2SW were repeat spawners with a 1‐year virgin sea age (*n* = 11, 1.9%). This finding is concordant with the low proportion of older MSW fish among several rivers studied here (Kusche et al., 2017) and does not preclude *vgll3* exhibiting consistent associations with advanced sea age among rivers in North America as observed in Norwegian populations. Surprisingly, we do not find evidence of allelic associations at *vgll3* in population‐level tests of association with 1SW proportion, despite it being identified with individual resequencing data. The lack of this association at the population level may be a result of the sex and region‐specific nature of this association, as well as the lack of known sea age classes in samples genotyped on the SNP array.

Using SNP array data, we found an association with river‐level 1SW proportion and *six6*, which covaried with population structure. The significant signal associated with population structure revealed in PCA, LFMM, and RDA analyses is also consistent with past associations of this locus with fine‐scale population structure and river‐specific features such as catchment area. This finding supports the possibility that this association may also correspond to a relationship with a correlated trait that is influenced by local environment rather than maturation itself (Pritchard et al., 2018; Zueva et al., 2021). This pattern reflects covariation between genetic architecture and population structure across regions exhibiting genome‐wide differentiation as revealed in PCA, as well as differences in 1SW proportion. GWA of individual‐level phenotypes and WGS data also failed to replicate this association in the present study, but we did observe differentiation at *six6* among northern and southern rivers which exhibit differences in 1SW proportions, as seen in allele frequency comparisons and PCA of WGS data. Together, these results indicate that *six6* itself may not be a major determinant of maturation across North American populations sampled here but is associated with broad‐scale variation in grilse proportion, also consistent with association with a correlated trait. In contrast, GWA of a European multigeneration aquaculture line uncovered significant associations in genomic regions near *six6* (Sinclair‐Waters et al., 2020), and this locus was also identified using haplotype‐based and single SNP association models in follow‐up analyses of wild individuals in Europe (Sinclair‐Waters et al., 2022), indicating a more direct role for *six6* in maturation among European populations compared to North America.

Our combined results indicate high context dependence of observed parallelism at *vgll3* and *six6*. These genes have shown associations with maturation timing and growth across the broader vertebrate lineage, indicating a conserved genetic role in control of maturation (Cousminer et al., 2013; Perry et al., 2014). Within salmonids, variable patterns of association have also been identified, with recurring associations with maturation across the lineage identified only at *six6* (Waters et al., 2021). However, we demonstrate that this relationship is variable even within Atlantic Salmon based on trans‐Atlantic divergence, sex, and regional population structure. Similar patterns of variable reuse of key genes implicated in intraspecific ecological divergence have also been observed in studies of *Pungitius* and *Gasterosteus* sticklebacks (Fang et al., 2021), as well as between *Timema* stick insect species (Villoutreix et al., 2020). Given the diversity of habitats colonized by Atlantic Salmon (Klemetsen et al., 2003), local environmental variation could play a role in biasing genetic parallelism by impacting the fitness landscape associated with trait/gene combinations. Environmental variation has been shown to significantly drive genome‐wide population structuring in Atlantic Salmon (Bradbury et al., 2014; Moore et al., 2014). Sea age has been shown to exhibit a strong latitudinal gradient in North America, with the lowest sea ages observed in Northern populations and associated with precipitation, river size, and lacustrine habitat access, as well as individual survival rates (Hutchings & Jones, 1998; Klemetsen et al., 2003). North American populations across Quebec and the Maritimes exhibit maturation ages more comparable to those in Norway, and we find that locations with lower 1SW proportions also exhibit SNP‐level parallelism with Northern European populations. Together, these findings indicate regional ecological conditions may impact selection for large‐effect loci. Future genomic studies may explore the relationship between life history, genomic, and environmental variation across the range as has recently been conducted in other systems, to quantify the level of overall genomic parallelism across traits and ecological contexts (Rennison et al., 2019).

### 4.2 Signals of polygenic adaptation

In addition to previously identified large‐effect loci, we uncovered nonparallel polygenic architecture underlying sea age variation across North American rivers. We build on recent detections of polygenic trait architecture underlying maturation detected within aquaculture (Mohamed et al., 2019; Sinclair‐Waters et al., 2020) and breeding experiments (Debes et al., 2021) by identifying sets of predictive loci for maturation. We achieved high prediction accuracy when accounting for population and sex‐level variation in per‐locus association with maturation and found nonparallel sets of loci enable accurate sea age prediction within regional groups. Consistent with other recent polygenic prediction studies in wild populations (Fuller et al., 2020; Hess et al., 2016; Lehnert, Kess, et al., 2019), our results highlight the utility of prediction approaches when accounting for genotype uncertainty and reveal a high capacity to predict sea age in wild Atlantic Salmon populations from genomic data.

The lack of overlap in the set of highly predictive SNPs used in each location and sex indicates high variability in the overall genetic architecture of sea age, consistent with polygenicity. Both variability in environmental conditions and drift likely impact this lack of parallelism, as maturation has been shown to be impacted by population‐specific genetic and environmental factors (Åsheim et al., 2023; Good & Davidson, 2016; Mobley et al., 2021). Atlantic Salmon exhibit high levels of environment‐driven genetic structuring, potentially altering the available set of standing adaptive variation across locations (Bradbury et al., 2014). Population structure has been shown to constrain rates of parallelism and promote heterogeneity in genetic architecture through stochastic loss of standing variation in other systems (Fang et al., 2021), and identification of high postcolonization drift in this and past studies is consistent with this process (Bradbury et al., 2014). Lastly, genome duplications may facilitate nonparallelism through diversification from duplicated gene copies (Ohno, 1970), and whole‐genome duplication within salmonids (Allendorf & Thorgaard, 1994; Lien et al., 2016) may have provided many genomic substrates for adaptation (Campbell et al., 2021). This variation in colonized environments, available adaptive variation, and the environmental variability of maturation together could drive variation in the individual genomic regions associated with maturation.

Our findings from gene‐set enrichment tests (Daub et al., 2013) reveal parallelism in pathways and molecular processes associated with maturation across the broader vertebrate lineage. Parallel enrichment at calcium signaling pathways is consistent with a significant role for calcium metabolism during oocyte maturation (Tosti, 2006) and osmoregulation changes during river spawning (Persson et al., 1998). Additional pathways also revealed significant associations with sexual maturation, including MAPK signaling, which plays a role in meiotic maturation (Kishimoto, 2003), phosphatidylinositol signaling associated with oocyte maturation, and previously in association with polygenic sea age variation in aquaculture fish (Hoshino et al., 2004; Mohamed et al., 2019), and gonadotropin releasing hormone (GnRH), implicated in male maturation (Wen et al., 2010). Neurological processes which may underlie necessary behavioral changes for both sexual maturation and migration (Mobley et al., 2021) were also enriched across comparisons, and we find enrichment in ErbB signaling, which has been linked to neural development and aggression (Barros et al., 2009), and phosphatidylinositol signaling, which has also been shown to mediate nervous system function (Raghu et al., 2019). Detection of significant enrichment among pathways implicated in cardiovascular function is also consistent with differences in energetic costs of large body size and migration in older sea winter fish (Jonsson et al., 1997) and elevated *vgll3* expression in heart tissue during maturation (Verta et al., 2020).

We find a high degree of parallelism at the pathway level despite no gene reuse across North American populations, consistent with increasing parallelism at higher levels of organization. Studies of parallelism have frequently identified high parallelism at the level of integrated traits that declines at finer scales of comparison (Bolnick et al., 2018). At the molecular level, recent investigations into parallelism in other salmonids (Jacobs et al., 2020) and humans (Bergey et al., 2018) have similarly uncovered a high degree of parallelism of enriched pathways despite low genetic overlap, suggesting that polygenic architectures underlying complex traits may map on to relatively limited molecular processes. Our results indicate largely idiosyncratic genomic changes at the gene and SNP level across regions and sexes contribute to a few shared higher‐level molecular pathways, corresponding to discrete differences in sea age. The observed molecular parallelism at the pathway level indicates that conserved processes with associations in other vertebrate lineages also underlie maturation in Atlantic Salmon. Interestingly, we find that this relationship holds despite large‐scale differences in 1SW proportions between sampled populations, as well as large variation in environmental conditions, and stock characteristics (e.g. growth rate, survival rate) associated with maturation age (Hutchings & Jones, 1998; Power, 1981). Our results indicate that despite significant plasticity in sea age and corresponding heterogeneity in genetic architecture (Ayllon et al., 2019; Besnier et al., 2023), the pathways implicated in maturation are repeatable between locations. These findings also indicate that ecological variation may play only a limited role in altering the set of conserved molecular functions associated with complex traits and suggest these functions may be predictable across both species and environmental contexts. Future research in this system may benefit from testing the generality of pathway level parallelism of maturation across European populations, as well as other traits, and salmonid and fish species.

### 4.3 Limitations and future directions

Our study provides the first genomic investigation into polygenic and molecular components of sea age variation in North American Atlantic Salmon, but follow‐up studies are necessary to build on uncertainty from limitations to the approaches used here. Our identification of different genes associated with sea age variation across individual‐level (WGS) and river‐level (SNP array) datasets indicates remaining sensitivity to population structure in analyses of population‐level phenotypic information. Replicability and informativeness of GWA studies can be significantly reduced when population structure is not appropriately controlled (Korte & Farlow, 2013); this challenge is exacerbated in instances of overlap between population structure and adaptation making population structure correction overly conservative in some instances (François et al., 2016). However, our individual‐level resequencing GWA showed only a weak association with major axes of population structure, and across methods, we uncovered previously identified large‐effect sea age loci, highlighting the utility of range‐wide sampling and individual phenotypes for GWA in wild populations. Our sample sizes here are comparable to those used in detection of sex‐specific large effect loci in Atlantic Salmon (Ayllon et al., 2015; Barson et al., 2015) and other salmonids (McKinney et al., 2021; Thompson et al., 2020), and in identification of polygenic patterns identified in gene‐set enrichment studies (Bergey et al., 2018; Foll et al., 2014). However, our study remains much smaller than large‐scale human and agricultural GWA studies, and follow‐up validation with larger, independent datasets and meta‐analysis of GWA scores will provide greater capacity to detect polygenicity in Atlantic Salmon using both GWA and gene‐set enrichment methods.

An additional potential source of uncertainty is that our WGS analyses were restricted to low‐depth (~3x) resequenced SNPs. Recent studies indicate significant roles for structural and epigenomic variation in mediating adaptation across many systems (Layton & Bradbury, 2022), and future assay of these types of variation may identify additional molecular mechanisms important in sea age variation. Low‐depth resequencing also introduces some uncertainty about variants detected. However, we employed large sample size (*n* > 500), and statistical control for genotype uncertainty through genotype likelihood approaches and imputation. Recent simulations have shown that with sample sizes, sequencing depths, and levels of population structure used here, both likelihood and imputation approaches should accurately capture allele frequencies (Lou et al., 2021), and we find very high and significant correlation among allele frequencies, *F*_ST_ from imputed data, and genotype likelihood GWA scores. However, as sequencing quality and depth improves, sequencing larger panels of individuals with technologies that capture more classes of variant (e.g., epigenomic modifications and structural variants) will aid in characterizing the genomic basis of sea age variation.

## 5 CONCLUSIONS

Adaptation of complex phenotypes such as life history variation often presents comparably complex underlying architectures. These traits may evolve through a combination of evolution from standing variation, hard and soft sweeps, and alleles of varying effect size. Here, we used GWA, genome scans, and random forest‐based polygenic prediction to explore the genomic basis of sea age variation in North American Atlantic Salmon. We found evidence of significant association with previously identified large‐effect genes and individual variants in *vgll3* as well as varying patterns of association based on both sex and region, suggesting both parallelism at large‐effect loci, and a high degree of genetic redundancy and polygenicity in this trait. Despite low overlap of the most strongly associated genes, we found a set of molecular pathways with conserved roles in maturation were consistently enriched among comparisons, revealing a core set of molecular mechanisms that underlie sea age variation in North American Atlantic Salmon. Our findings demonstrate clear pathways and genes for future investigations of maturation traits, and show how methods aimed at resolving polygenic patterns can uncover the molecular basis of a complex phenotype with vary genetic architectures across wild populations.

## AUTHOR CONTRIBUTIONS

**Tony Kess:** Conceptualization (lead); data curation (lead); formal analysis (lead); investigation (lead); methodology (lead); project administration (supporting); software (lead); validation (lead); visualization (lead); writing – original draft (lead); writing – review and editing (lead). **Sarah J. Lehnert:** Data curation (lead); resources (equal); validation (equal); writing – original draft (equal); writing – review and editing (equal). **Paul Bentzen:** Data curation (supporting); funding acquisition (supporting); investigation (equal); methodology (supporting); project administration (supporting); resources (equal); supervision (equal); writing – original draft (equal); writing – review and editing (equal). **Steven Duffy:** Conceptualization (lead); data curation (lead); methodology (lead); project administration (lead); resources (lead); supervision (lead); writing – original draft (equal); writing – review and editing (equal). **Amber Messmer:** Conceptualization (equal); data curation (equal); methodology (lead); project administration (equal); resources (equal); writing – original draft (equal); writing – review and editing (equal). **J. Brian Dempson:** Conceptualization (equal); data curation (equal); investigation (equal); resources (equal); writing – original draft (equal); writing – review and editing (equal). **Jason Newport:** Formal analysis (equal); methodology (equal); software (equal). **Christopher Whidden:** Formal analysis (equal); methodology (equal); software (equal); validation (equal); writing – original draft (equal); writing – review and editing (equal). **Martha J. Robertson:** Data curation (equal); resources (equal); writing – original draft (equal); writing – review and editing (equal). **Gerald Chaput:** Data curation (equal); methodology (equal); resources (equal); validation (equal); writing – original draft (equal); writing – review and editing (equal). **Cindy Breau:** Data curation (equal); methodology (equal); resources (equal); writing – original draft (equal); writing – review and editing (equal). **Julien April:** Data curation (equal); methodology (equal); resources (equal); writing – original draft (equal); writing – review and editing (equal). **Carole‐Anne Gillis:** Data curation (equal); methodology (equal); resources (equal); writing – original draft (equal); writing – review and editing (equal). **Matthew Kent:** Data curation (equal); methodology (equal); resources (equal); writing – original draft (equal); writing – review and editing (equal). **Cameron M. Nugent:** Formal analysis (supporting); methodology (supporting); software (supporting); validation (supporting); writing – original draft (equal); writing – review and editing (equal). **Ian R. Bradbury:** Conceptualization (lead); data curation (equal); formal analysis (supporting); funding acquisition (lead); investigation (lead); methodology (equal); project administration (lead); resources (lead); supervision (lead); validation (supporting); visualization (supporting); writing – original draft (lead); writing – review and editing (lead).

## CONFLICT OF INTEREST STATEMENT

The authors declare no conflict of interest.

## Supporting information


Figures S1–S7

Tables S1–S7

## Data Availability

SNP array genotypes in plink format and phenotype data for association analyses are available on dryad at https://doi.org/10.5061/dryad.g1jwstqz3. Raw reads from WGS data have been uploaded to the NCBI SRA with accession number PRJNA1083490. All scripts used for analysis in this study are available at: https://github.com/TonyKess/seaage_GWAS.
