# Supplementary material for: Variable parallelism in the genomic basis of age at maturity across spatial scales in Atlantic Salmon
Source: Ecol Evol. 2024 Apr 5;14(4):e11068. doi: 10.1002/ece3.11068 (PMC10995719; doi:10.1002/ece3.11068)

**Supplementary Figure 1. Scree plot of variation explained by PCs at K = 10 from PCA of individuals genotyped on the SNP array using *pcadapt.***


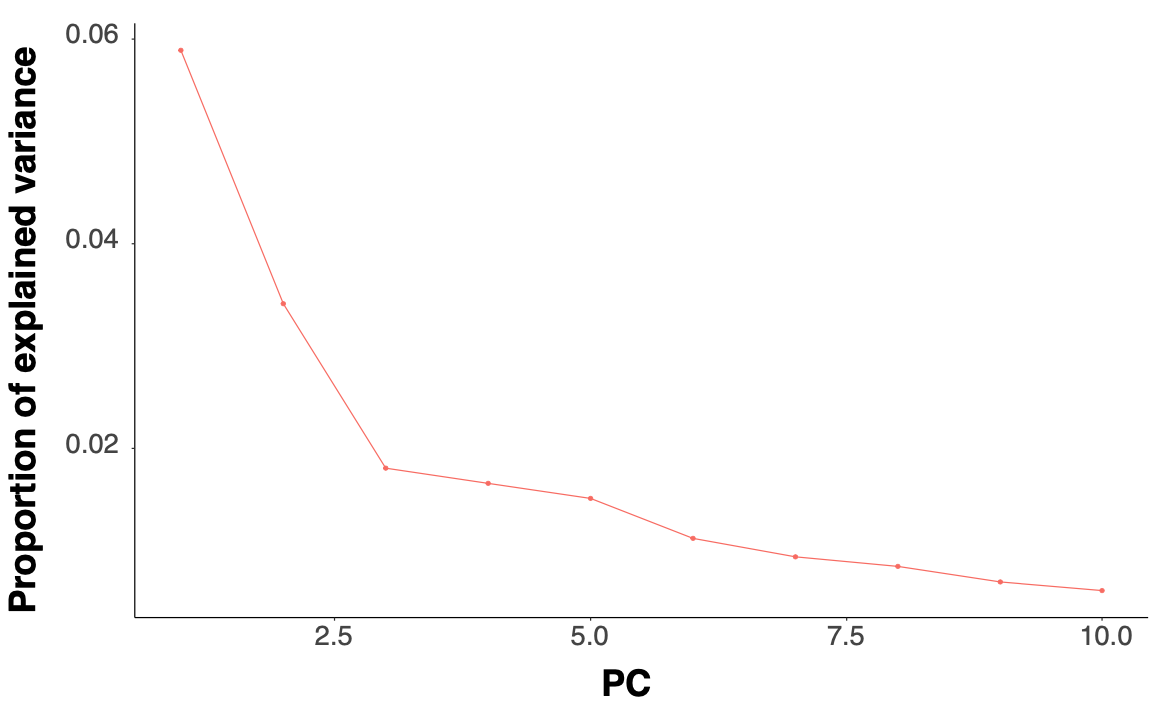


**Supplementary Figure 2 Cross-entropy criteria across K ancestral groups estimated from individuals genotyped on the SNP array using *snmf.***


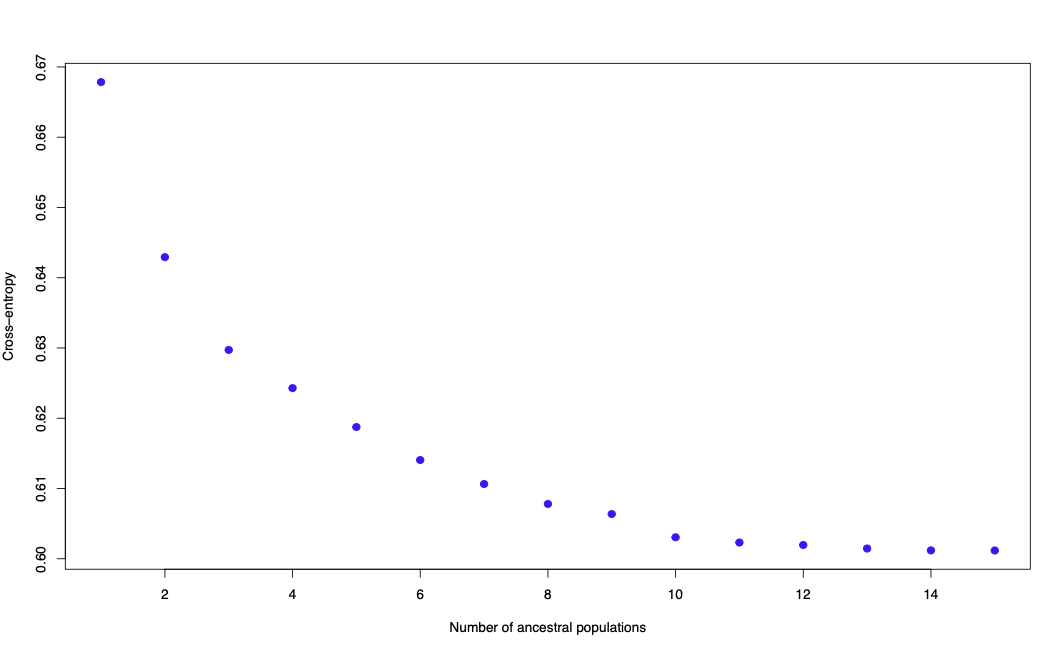


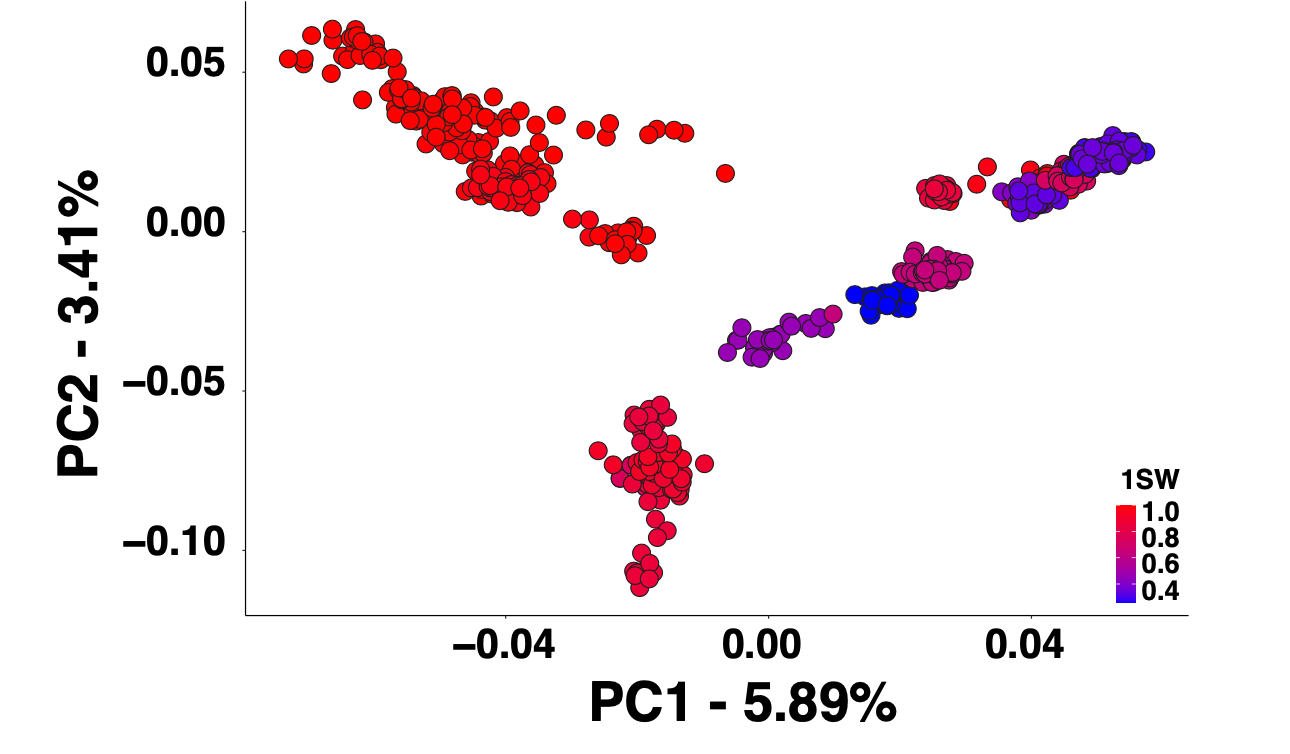
**Supplementary Figure 3. Population structure inferred from principal component (PC) analysis in *pcadapt* of individuals genotyped on the SNP array, coloured by 1SWz proportion in region of origin.**

**Supplementary Figure 4. Allele frequencies at top scoring SNP in six6 from PCANGSD and, and top scoring SNP vgll3 and Sinclair-Waters et al. 2022 in GWA.**


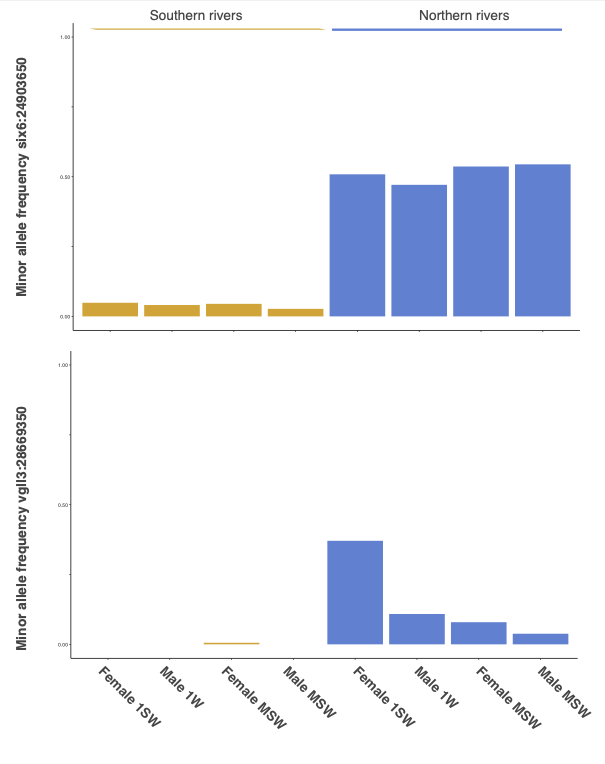


**Supplementary Figure 5. -log10 transformed qvalues from GWA of sea age of all samples with population structure correction with the first two PCs estimated in PCANGSD, restricted to the 50kb window surrounding**
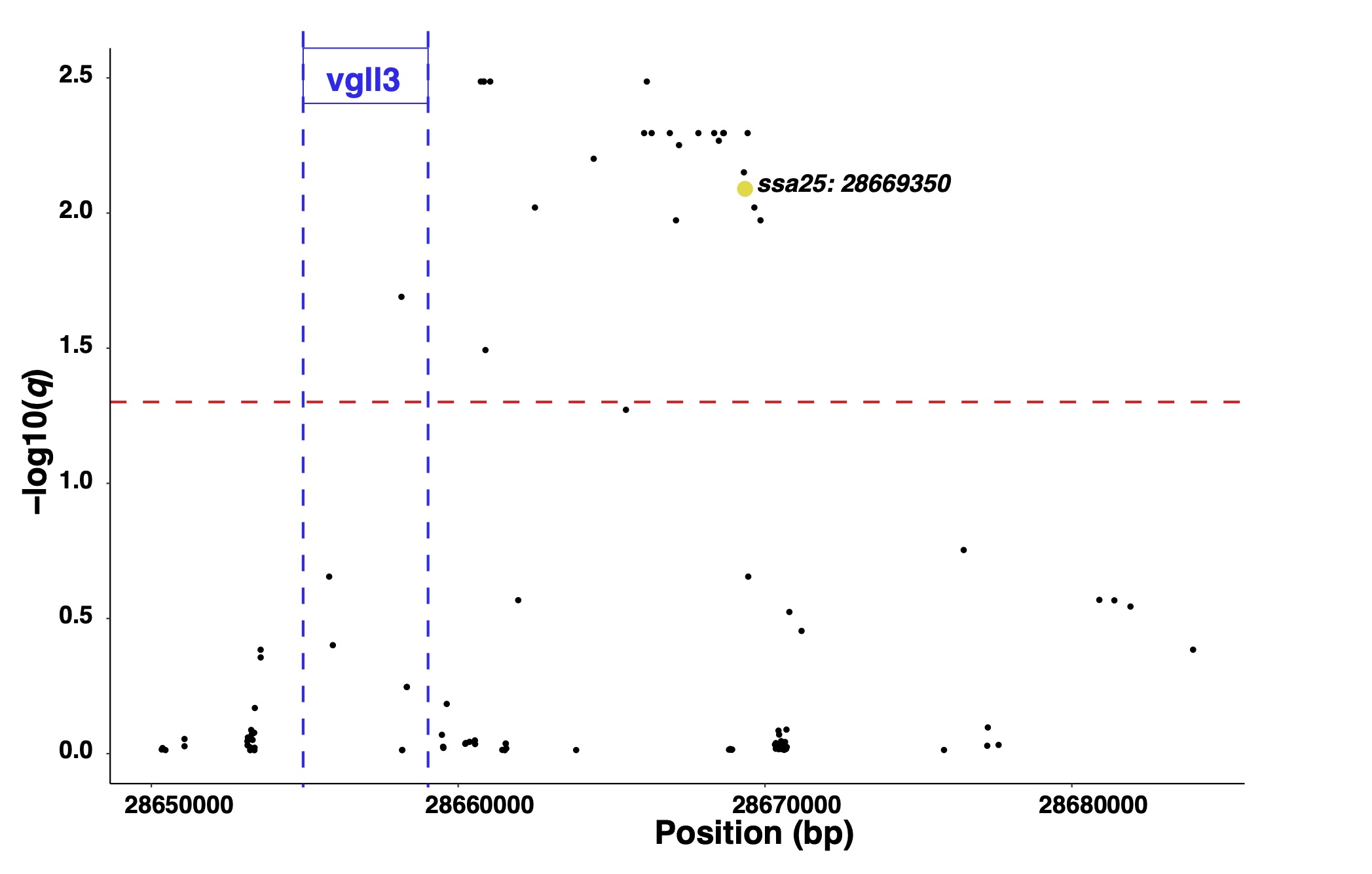
***vgll3.***

**Supplementary Figure 6. Receiver operating characteristic (ROC) curve comparing sensitivity and specificity of random forest prediction of sea age from likelihood adjusted genotype dosages at the top 500 sea age associated SNPs genotyped in 582 individuals.**


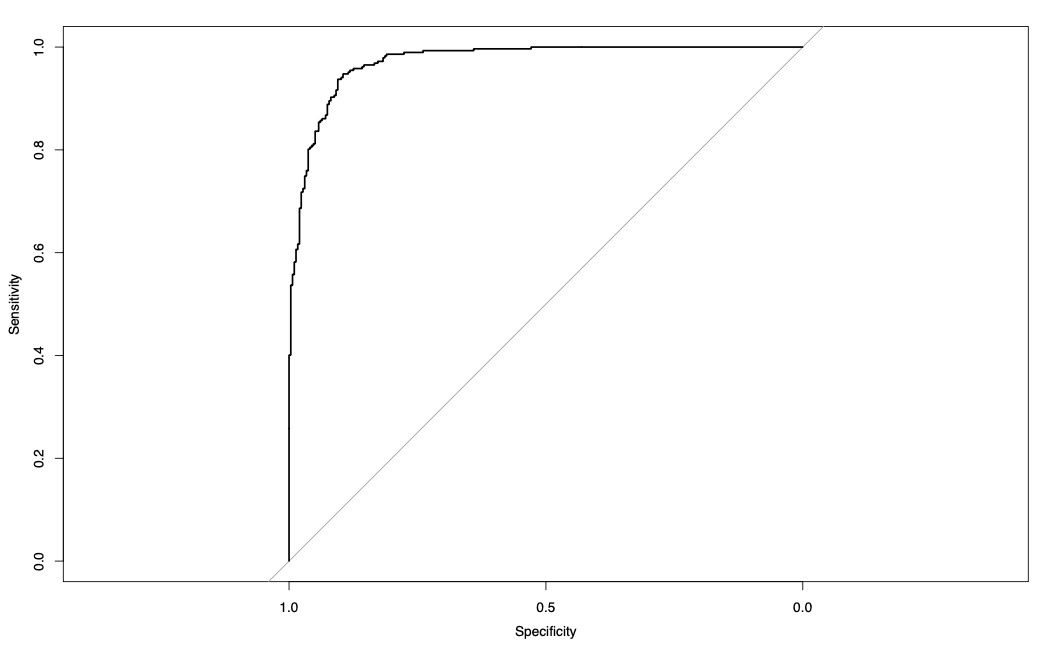

Supplement: Supplementary file 1 — Figures S1–S7 [file ECE3-14-e11068-s002.docx]
